# Chemobrain and Cancer Survivorship: A Scoping Review of the Literature

**DOI:** 10.1002/cam4.71383

**Published:** 2025-11-12

**Authors:** Michael J. Rovito, Khushi M. Chauhan, Humnah Baig

**Affiliations:** ^1^ Department of Health Sciences University of Central Florida Orlando Florida USA; ^2^ University of Central Florida Orlando Florida USA

**Keywords:** advocacy, chemobrain, quality of life, survivorship

## Abstract

**Background:**

Cancer‐related cognitive impairment (CRCI), commonly known as chemobrain, is characterized by deficits in memory, processing speed, and executive function. While traditionally linked to chemotherapy, emerging evidence suggests that hormone and radiation therapies may also contribute. Despite its high prevalence, CRCI remains underrecognized in oncology research and clinical practice, limiting the development of targeted interventions. Estimates suggest that up to 75% of cancer patients experience cognitive impairment during treatment, with approximately 35% reporting persistent symptoms months or even years post‐treatment. However, much of the existing literature has focused on breast cancer, leaving a critical gap in understanding CRCI in other cancer populations.

**Aims:**

This scoping review systematically analyzed research on CRCI across various malignancies, excluding breast cancer, to provide a broader perspective.

**Materials & Methods:**

A comprehensive literature search identified 29 studies examining cognitive impairment in patients with colorectal, testicular, lung, prostate, and hematological cancers.

**Results:**

Findings suggest that the severity and duration of cognitive decline vary depending on cancer type and treatment modality, with some therapies inducing transient symptoms and others contributing to prolonged deficits. Existing cognitive assessment tools, such as the Mini‐Mental State Examination (MMSE), often lack the sensitivity to detect subtle neurocognitive changes, highlighting the need for more precise diagnostic measures. Methodological challenges, including small sample sizes and reliance on self‐reported cognitive symptoms, further hinder the characterization of CRCI in non‐breast cancer populations.

**Discussion:**

Given the increasing recognition of CRCI across diverse malignancies, future research should prioritize longitudinal studies, advanced neuroimaging techniques, and targeted interventions to mitigate cognitive impairment. A more comprehensive understanding of CRCI in various cancer populations is essential for refining clinical guidelines, improving cognitive assessments, and enhancing survivorship care.

**Conclusion:**

Expanding research efforts beyond breast cancer will help optimize supportive care strategies and improve the quality of life for individuals affected by cancer‐related cognitive dysfunction.

## 1 Introduction

### 1.1 Chemobrain

Chemotherapy‐induced cognitive impairment (CICI), most notably labeled *chemobrain*, is characterized by memory deficits, including memory impairments, reduced processing speed, and executive dysfunction, which can negatively impact patients' HRQoL [1, 2]. Chemobrain is a misnomer as it is not only caused by chemotherapy but also by other cancer treatments (e.g., select hormone therapies and radiotherapy), or even by the cancer itself [3]. While the underlying causes of chemobrain remain unclear, research shows that host characteristics, immune dysfunction, neural toxicity, and genetics may contribute significantly to its development [4]. Furthermore, the severity of cognitive impairment has been associated with dosage levels [5].

Although the “chemobrain” term is commonly used in both research and clinical settings, it is increasingly recognized that cognitive changes occur not only from chemotherapy but also from other cancer‐related factors, including the disease process itself and other treatments. Throughout this review, therefore, we use the broader term “cancer‐related cognitive impairment” (CRCI) to encompass these overlapping operational definitions. Simultaneously, we acknowledge that many of the included studies specifically reference chemotherapy‐induced effects.

### 1.2 CRCI Significance

A study by Tilsed [6] reported that 75% of cancer patients experience CRCI during treatment, with 35% continuing to experience symptoms several months post‐treatment. According to the American Cancer Society (ACS) [7], in 2022, there were 18.1 million cancer survivors, suggesting that more than six million individuals may currently be experiencing CRCI. Research suggests that in some patients, CRCI may have a delayed onset that may persist for up to 20 years of treatment [8]. Despite its widespread impact on cancer patients, CRCI has been historically underrecognized in medical research and clinical practice [9]. Clinicians often attribute CRCI symptoms to other chemotherapy side effects, such as fatigue and neuropathy, potentially contributing to its persistent underreporting [10].

### 1.3 CRCI and the Focus on Breast Cancer: Gaps in the Literature

While CRCI is a phenomenon that occurs in patients with all types of cancer, a large share of existing research highlights its impacts on breast cancer survivors. Studies report that over 50% of breast cancer survivors do in fact experience cognitive dysfunction symptoms [11], highlighting its significance within this patient population. Notably, breast cancer research receives the highest level of funding among individual cancer types [12], enabling extensive work in prevention, awareness initiatives, and investigative survivorship research. However, the emphasis on CRCI in breast cancer research should not overshadow its impact on patients with other cancer types.

According to the Division of Cancer Control and Population Sciences of the National Institute of Health (NIH) [13], the number of cancer survivors is projected to increase to 22.5 million by 2032. As the number of cancer survivors rises, so do issues related to the disease and its treatment [14] (i.e., CRCI). Enhancing our knowledge of CRCI across diverse types of cancers is crucial for developing comprehensive post‐care for treatments and recommendations that cater to specific patient needs.

To the best of our knowledge, no comprehensive review has synthesized research on CRCI across multiple cancer types in a single scoping review. Prior investigations have disproportionately focused on breast cancer, resulting in a narrow understanding of CRCI that may not fully capture its prevalence, severity, and underlying mechanisms in other malignancies. This focus, if continued, could possibly contribute to a future research gap that may limit clinical awareness, hinder early identification, and restrict the development of tailored interventions for diverse cancer populations. Given that cognitive impairment can significantly affect treatment adherence, daily functioning, and overall quality of life, a broader examination of CRCI across cancer types is essential.

This review seeks to address this critical gap by systematically analyzing studies that assess CRCI in colorectal, testicular, lung, prostate, and hematological cancers. By consolidating evidence across these malignancies, we aim to provide a more comprehensive understanding of how cancer therapies contribute to cognitive decline beyond the breast cancer paradigm within the methodological context of a scoping review. In doing so, we hope to lay the groundwork for future research that refines cognitive assessment tools, advances neuroimaging techniques, and informs the development of evidence‐based interventions. Expanding the scope of CRCI research is crucial to optimizing survivorship care, enhancing clinical guidelines, and ultimately improving long‐term outcomes for individuals affected by cancer‐related cognitive dysfunction.

## 2 Methods

### 2.1 Study Design

A scoping review of the literature was conducted to identify existing research on CRCI across all cancer types, excluding breast cancer. The goal of this research was to understand the depth and breadth of available research on CRCI outside of breast cancer studies. In adhering to the PRISMA‐ScR (Preferred Reporting Items for Systematic Reviews and Meta‐Analyses Extension for Scoping Reviews) reporting guidelines, we ensured transparency and rigor in the review process by detailing our inclusion and exclusion criteria, search strategy, screening process, and data extraction methods. This systematic approach enabled us to synthesize the findings comprehensively, offering insights into CRCI among all cancer sites.

### 2.2 Data Sources and Search Strategy

The search strategy used the Ovid Medline (1950 to present), CINAHL (1982 to present), PsycINFO (1806 to present), All EBM Reviews (2024), Ovid Healthstar (1966 to present), ERIC, and Google Scholar (2024) databases to locate relevant literature. Further, the review utilized ancestry and gray literature searches to ensure full capture of relevant behavioral intervention research. All databases except for Google Scholar and ERIC used OVID Gateway. Google Scholar used its own search catalog while ERIC employed EBSCOhost.

### 2.3 Inclusion/Exclusion Criteria

This review of literature includes peer‐reviewed, English‐language analytical design trials that examine the prevalence of cognitive performance measurements among cancer patients. Studies focusing exclusively on breast cancer were excluded. Studies assessing cognitive impairment, self‐reported cognitive dysfunction, patient preferences, and clinical outcomes were included to provide a comprehensive evaluation of CRCI. To assess the efficacy of diagnoses and produce a best‐practices model among cancer survivors, only academic journals that experimentally assessed the relevance of CRCI were reviewed.

### 2.4 Screening Procedure

#### 2.4.1 Primary Screening

Our first assessment screened titles and abstracts for relevance. Articles mentioning cognitive function and cancer were included at this stage, encompassing those referring to CRCI.

#### 2.4.2 Secondary Screening

In the secondary screening, full‐text articles were assessed for relevance. Animal, lab‐based studies were excluded, as well as non‐peer‐reviewed and/or non‐published material (inclusive of gray literature). The articles kept were human subject behavior‐based studies.

#### 2.4.3 Tertiary Screening

Our third assessment eliminated any studies mentioning breast cancer. Interventions were excluded if they solely considered breast cancer populations. If other cancers were mentioned in a study where breast cancer was discussed, it was still included.

### 2.5 Study Selection

**FIGURE 1:**
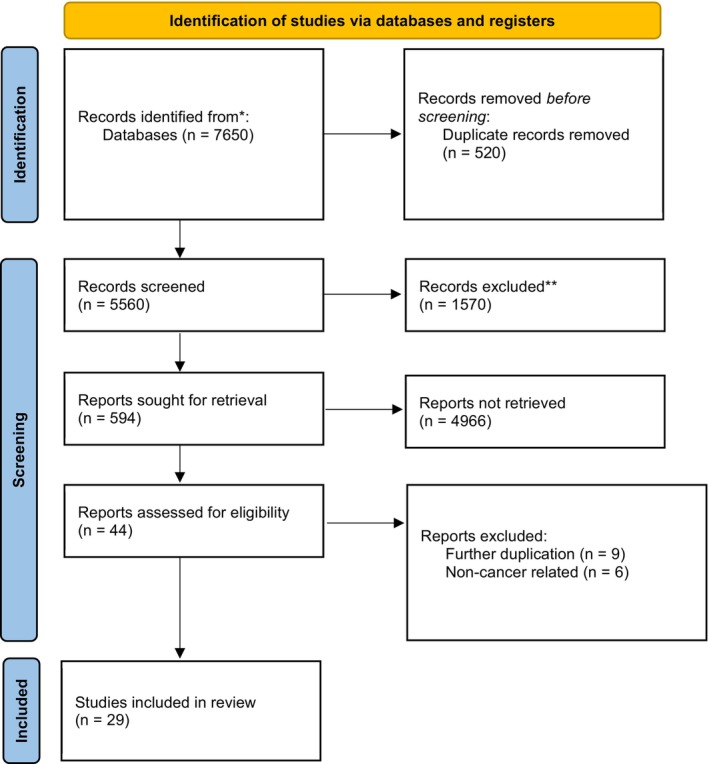
PRISMA flowchart.

The initial database search yielded 7650 articles retrieved from the initial search, and after publication date, title, and abstract screening, 44 articles were retrieved for full‐text review. Following duplicate removal and eligibility screening, 29 studies met the inclusion criteria for synthesis (see Figure 1). We included studies assessing the association between CRCI and cancer, explicitly excluding those focused solely on breast cancer. Through systematic analysis of 29 studies, we explored variations in CRCI reporting across different cancer types. The extracted information was used to analyze the scope of CRCI research beyond breast cancer. The 29 articles were published between 1995 and 2021, with nine studies being published in the last five years.

## 3 Results

### 3.1 Summary

The studies reviewed displayed considerable heterogeneity in design, sample size, and methodological rigor. Sample sizes ranged from small pilot cohorts of fewer than 30 participants to national surveys exceeding 10,000 respondents. Cancer types spanned solid tumors (e.g., breast, colorectal, lung, prostate, and testicular) and hematologic malignancies, with the majority of research focused on breast and colorectal cancer populations. Treatment exposures varied in duration and intensity, from single‐agent regimens to multi‐cycle adjuvant chemotherapy and adjunct hormonal or radiation therapies. Cognitive assessment methods included objective neuropsychological testing, computerized tasks, and self‐report measures such as FACT‐COG, each with differing sensitivity to subtle changes. Recruitment was primarily clinic‐based, though several studies drew from population registries or survivorship cohorts. This variability underscores the absence of standardized CRCI assessment protocols and complicates direct cross‐study comparisons, reinforcing calls for consensus measures and consistent reporting of treatment‐related variables.

### 3.2 Cancer Type

Of the 29 articles (see Table 1), 11 did not specify a single cancer type or included multiple types of cancer, including breast cancer. Among these 11 studies, five reported that breast cancer patients constituted the majority of their sample. For example, Jansen [24] and colleagues reported that they had a 58% breast cancer sample, followed by 18% hematological malignancies. Similar results were reported by Khan et al. [27], who had a sample of 73 breast cancer patients (51%) and 69 colorectal cancer patients (49%). Schmidt [14] and collaborators documented that the most common types of cancer reported were breast cancer (29.1%), testicular (9.1%), prostate (7.4%), etc. (2016). Additionally, Wazqar [36] found that participants with breast cancer made up (34%) of the sample, followed by gastrointestinal cancer (24%). Kohli et al. [39] also found that the most common diagnosis among their 595 participants was breast cancer (*n* = 320, 54%). Of these studies where breast cancer was so prevalent in mixed samples, no subgroup analyses were provided.

**TABLE 1:** Characteristics of studies included in scoping review.

References	Design	Sample (*n*)	Type/s of cancer	Primary findings	Limitations	Year
Effects of long‐term androgen deprivation therapy on cognitive function over 36 months in men with prostate cancer [15]	Longitudinal Observational Study	*n* = 241	Prostate	A global summary of cognitive change found no statistically significant worsening of cognitive function among ADT users compared with controls. The ongoing use of ADT for up to 36 months does not appear to be associated with cognitive decline	Did not include men who were being treated with intermittent ADT, antiandrogen monotherapy, or newer hormonal agents such as abiraterone and enzalutamide. There was also no correction for multiple comparisons	2016
Lack of a chemobrain effect for adjuvant FOLFOX chemotherapy in colon cancer patients. A pilot study [16]	Pilot Study	*n* = 57	Colorectal	There was no effect on cognitive function related to chemotherapy found. Additionally, there was no statistical significance and clinically relevant effect on the global cognitive functions of this standard‐of‐care adjuvant chemotherapy for colon cancer	The authors are not able to completely explain our data; they cannot argue that the total dose of oxaliplatin can have a certain slight detrimental even transient effect on cognitive function and depression. Also, the study was conducted on a small population	2012
Either called “chemobrain” or “chemofog,” the long‐term chemotherapy‐induced cognitive decline in cancer survivors is real [17]	Systematic Review	70 articles were included	Solid tumors	Findings indicate that CICI is a relatively common event that, in most cases, remains underdiagnosed, thereby adversely affecting the quality of life of patients with cancer	The small samples, most likely leading to reduced power, pose a significant methodological limitation of the available studies, thereby bolstering the need for larger long‐term longitudinal studies to accurately assess the importance of neuroimaging changes in cancer survivors and their relationship with CICI	2011
Cognitive impairment in testicular cancer survivors 2 to 7 years after treatment [18]	Observational cross‐sectional cohort study	*n* = 72	Testicular	In group‐level analyses, survivors exhibited significantly impaired scores on a majority (9/12) of the neuropsychological outcomes (*p* < 0.01). In individual‐level analyses, 62.5% of the survivors were classified as having CI, significantly exceeding the expected normative frequency of 25% (binomial test: *p* < 0.001)	Did not have direct access to information about the premorbid cognitive status of the group. Also, the study only included tests with available age‐adjusted. There was recruitment bias as only one‐third (31.2%) of the total number of eligible survivors identified for the present study agreed to participate. And, heterogeneity of clinical variables such as time since treatment, treatment modalities, cancer type, and stage may limit the generalizability of the results	2015
Cognitive deficits in patients with small cell lung cancer before and after chemotherapy [19]	Prospective study	*n* = 46	Lung	The study confirmed previous reports of a high incidence of cognitive impairment in SCLC patients before they received PCI. Further, these deficits do not seem to be related to chemotherapy, and they are present in patients who are newly diagnosed and have not yet been treated	Small sample size and it is an older study	1995
Longitudinal study of cognitive dysfunctions induced by adjuvant chemotherapy in colon cancer patients [20]	Longitudinal study	Pre‐chemotherapy (*n* = 81), post‐chemotherapy (*n* = 73), and after 6‐month follow‐up (*n* = 54)	Colon	Adjuvant FOLFOX4 in patients with colon cancer can have a negative effect on verbal memory. This deterioration is usually mild and transient	This is a relatively small series, with an important attrition rate, where practice effects may mask some deficits	2014
Perceived cognitive impairment in people with colorectal cancer who do and do not receive chemothearpy [21]	Longitudinal Study	*n* = 362	Colorectal	No association was seen between total FACT‐COG or PCI, and neuropsychological domains. A weak‐moderate association was found between PCA and attention/executive function and visual memory	Attrition and missing data and an over‐representation of women in the control group. Also, there remains no commonly accepted definition of what constitutes a cognitive symptom or a cut‐off score for FACT‐COG or its subscales	2018
Living with cognitive changes following completion of cancer treatment [22]	Transition Study	*n* = 13,258	Breast, Prostate, Colorectal, and Melanoma diseases with no metastatic spread, and selected Hematological cancers	Almost 40% of the survivors in this study reported concerns about cognitive changes	There could be some sampling bias. The survey was distributed to a randomly selected sample of cancer survivors from 10 Canadian provinces. However, there could be inherent biases in the selection process. The data collected heavily relies on self‐reporting by the respondents. This introduces the possibility of recall bias, where respondents may not accurately remember their experiences, especially regarding cognitive changes that might have occurred sometime in the past	2020
Cognitive functioning and quality of life in long‐term adult survivors of bone marrow transplantation [23]	Retrospective Study	*n* = 40	Hematological	These data indicate that Bone Marrow Transplants may lead to cognitive complaints and late cognitive deficits in long‐term adult survivors. Cognitive functioning should therefore be used as an outcome parameter in BMT studies	The sample size is relatively small due to the low survival rates and the limited number of disease‐free survivors. Another limitation is that all patients in our study received a conditioning regimen with high‐dose chemotherapy and TBI and the effects directed to TBI only were not assessed. In addition, with this sample size, we were unable to assess whether specific chemotherapeutic agents or dosages affect cognitive functioning. Similarly, the retrospective design and lack of pretreatment baseline assessment preclude definite conclusions about a change in cognitive functioning over time	2002
A meta‐analysis of studies of the effects of cancer chemotherapy on various domains of cognitive function [24]	Meta‐Analysis	16 studies that evaluated cognitive function in chemotherapy patients were included in the study	Breast cancer (58%), followed by Hematologic malignancies (18%), mixed diagnoses (10%), Lung cancer (9%), and Lymphoma (5%)	Only one domain of cognitive function (i.e., visual memory) had significant chemotherapy‐induced impairment across all comparison types. Data from this meta‐analysis supported the hypothesis that chemotherapy can have a negative impact on cognitive function. However, most deficits in this study ranged from small to moderate and were nonsignificant	The paper did not provide specific data on the effects of chemotherapy compared with other cancer treatments. Another limitation of this meta‐analysis was that information was not provided on how various neuropsychologic tests were categorized in terms of domains of cognitive function. Several neuropsychologic tests, used in the 30 studies evaluated, were excluded from analysis without any explanation	2005
Impact of Lung Cancer Treatment on Cognitive Functioning, Clinical Lung Cancer [25]	Systematic Review	39 longitudinal articles were included	Lung	There seems to be a significant negative effect on the attention and memory subdomain and very likely also on fluency, in patients with SCLC receiving PCI after chemotherapy, surgery, and thoracic radiotherapy. Therefore, it is debatable whether treatment with PCI is wise in patients with significant pre‐existing cognitive dysfunction	There was diversity in study populations concerning performance status, age, gender, tumor stage, timing of cognitive assessments, and the instruments used to assess cognitive functioning. Limiting the generalizability of the results. There was a large amount of studies with a high loss of follow‐up	2020
Health‐related quality of life and self‐reported cognitive function in patients with delayed neurocognitive recovery after radical prostatectomy: a prospective follow‐up study [26]	Prospective Study	*n* = 367	Prostate	Delayed neurocognitive recovery in the early period after radical prostatectomy has a long‐term impact on patients' daily lives by impairing memory, attention, action, and perception. Delayed neurocognitive recovery in the early postoperative period was significantly associated with self‐reported cognitive failures (B for no DNCR = −0.411 [95% CI: −0.798;0.024], *p* = 0.038), but not with physical (B = 0.082 [95% CI: −0.021;0.186], *p* = 0.118) or mental HRQoL (B = −0.044 [95% CI: −0.149;0.062], *p* = 0.417) 12 months after surgery	The study protocol was initially designed to detect a difference in the incidence of DNCR between robot‐assisted radical prostatectomy and open retropubic surgery. The study did not assess cognitive failures and HRQoL preoperatively. Therefore, we cannot evaluate a potential change in HRQoL from preoperative to postoperative values. We had information about neoadjuvant ADT but not about whether adjuvant ADT was administered. Both of these can impact postoperative HRQoL and are usually associated with a more advanced tumor stage	2021
Immediate‐term cognitive impairment following intravenous (IV) chemotherapy: a prospective pre‐post design study [27]	Prospective pre‐post design study	*n* = 142	73 breast, 69 colorectal	Psychomotor vigilance test reaction time slowed significantly immediately post‐chemotherapy compared to a pre‐chemotherapy baseline, and levels of impairment similar to the effects of alcohol consumption in other studies were seen in 40% of patients	Small study populations, heterogeneity, and the presence of confounding variables limit the interpretation of data regarding chemotherapy and cognition	2019
Effects of Adjuvant Chemotherapy on Cognitive Function of Patients With Early‐stage Colorectal Cancer [28]	Prospective Study	*n* = 57	Colorectal	Patients with CRC who received adjuvant 5‐fluorouracil with or without oxaliplatin presented with a decline in executive function after 12 months compared with patients with localized disease who had not received chemotherapy	The study was limited by the small sample size (*n* = 57), the lack of a control group, and the brief cognitive assessment used	2019
Cognitive and Brain Structural Changes in a Lung Cancer Population [29]	Cross‐sectional study	*n* = 28	Lung	Lung cancer patients exhibit cognitive impairment before and after chemotherapy	The cross‐sectional design of the study may have limited the possibility of clearly isolating the effect of chemotherapy from more general cancer‐related changes	2015
The impact of cancer treatment on cognitive efficiency: Chemobrain–does it exist? [30]	Case–Control Study	*n* = 68	Lung	In the people diagnosed with a malignant disease, subject to chemotherapy, it was possible to observe significant deterioration of cognitive efficiency in the scope of information processing speed, working memory, executive functions, and verbal fluency. No statistically significant differences in cognitive functioning were found between subsequent stages of the examination	The small size of the studied groups, as well as the disproportions in the demographic distribution of study participants in each of them (gender), are limitations	2020
Long‐Term Cognitive Functioning in Testicular Germ‐Cell Tumor Survivors [31]	Prospective Study	*n* = 155	Testicular	Of the total survivors, 138 received treatment beyond orchiectomy, and 17 controls had orchiectomy alone. Any treatment resulted in significantly greater cognitive difficulties on the overall cognitive function score	A limitation is a smaller number of patients in some subgroups, which probably prevented the achievement of statistical significance in several attributes. Another limitation is an imbalance in age, which could have contributed to changes in the cognition of survivors	2018
Predictors of cognitive decline in people with cancer undergoing chemotherapy, European Journal of Oncology Nursing [32]	Cross‐sectional study	*n* = 175	Various types of cancers	Because of the limited sensitivity of the MMSE scale for detecting more subtle changes in cognitive ability (Prabhu et al., 2014), future studies should use updated instruments that are more sensitive for this purpose	Old age and cumulative chemotherapy cycles were the main influential factors for objectively confirmed cognitive decline, and fatigue was the most common predictor of self‐reported cognitive decline. Depression was one of the predictors of perceived cognitive decline, but it was not significant for objectively measured cognitive function. Thus, treatment‐related factors such as fatigue had a greater impact on cognitive decline than psychological factors	2017
Changes of Cognitive Function and Fatigue following Chemotherapy in Patients with Gastrointestinal Cancer: A Prospective Controlled Study [33]	Prospective Controlled Study	*n* = 133	Colorectal and stomach	Chemotherapy was associated with increased cognitive decline and fatigue in cancer patients with cancer	Longitudinal studies need to be done to see the effects of chemotherapy on other cancers	2019
Prevalence of perceived cognitive dysfunction in survivors of a wide range of cancers: results from the 2010 LIVESTRONG survey [14]	A cross‐sectional study	*n* = 3108	Various types of cancer	Current perceived cognitive dysfunction was reported by nearly half of respondents (45.7%), across a wide range of cancer types, with the highest prevalence among survivors of central nervous system cancers. Receiving chemotherapy and the current report of depressive symptoms were both strongly associated with current perceived cognitive dysfunction	First, the study relied on a cross‐sectional, anonymous survey. Second, the survey did not employ validated self‐report measures of depression or cognitive function. Third, cognitive dysfunction was not assessed objectively using well‐established (though lengthy) cognitive testing approaches. Fourth, two of the items used to assess PCD (“I have had difficulties doing activities that require concentration” and “I have been bothered by having a short attention span”) overlap with one of the diagnostic criteria for depression (DSM‐V, APA)	2015
Characteristics associated with inter‐individual differences in the trajectories of self‐reported attentional function in oncology outpatients receiving chemothearpy [34]	Cross‐sectional study	*n* = 1329	Breast, gastrointestinal (GI), gynecologic (GYN), or lung cancer	Prior to their next dose of CTX, patients reported moderate levels of attentional function that persisted over two cycles of CTX. Many of the clinical and symptom characteristics associated with decrements in attentional function are amenable to interventions	The evaluation of cognitive function was limited to a self‐report measure that primarily evaluated changes in executive function	2016
Cognitive Function in Patients With Colorectal Cancer Who Do and Do Not Receive Chemotherapy: A Prospective, Longitudinal, Controlled Study [35]	Prospective, Longitudinal, Controlled Study	*n* = 239	Colorectal	Patients with CRC had substantially more cognitive impairment at every assessment than HCs, with no significant added effect of chemotherapy. Mechanisms of cognitive impairment remain unknown	Limitations of our study are that our HCs were not well balanced for sex or primary language and were not assessed at 24 months. As in all longitudinal studies, data were missing for some patients with cancer	2015
Cognitive Dysfunction and Its Predictors in Adult Patients With Cancer Receiving Chemotherapy: A Cross‐Sectional Correlational Study [36]	Cross‐sectional Correlational Study	*n* = 100	Various types of cancers	The data showed that the participants experienced moderate‐to‐severe cognitive dysfunction. Participants performed poorly in the divided attention and memory cognitive domains. Age, educational level, and depression factors were found to be significant predictors of cognitive dysfunction	The convenience sample of 100 adult patients with cancer in one geographic location (Jeddah, SA) reduces the generalizability of the findings. A second limitation of this study was the use of a cross‐sectional design only at one point in time and without a control group. A third limitation was the lack of current studies in the field of oncology nursing on this topic in SA to allow for a direct comparison of findings	2019
Cognitive effects of chemotherapy: An integrative review [37]	Integrative Review	79 articles	Various types of cancers	Thematic analysis identified four broad themes within the literature regarding chemotherapy induced cognitive impairment. Identified themes included; cognition as part of a complex scenario, proof of existence and searching for the cause, learning to play the game and timing of cognitive impairment	There could have been selection bias because the article states that articles were included in the review if they focused on cognitive function or cognition relating to quality of life, involved solid malignancies, and were published in English. This inclusion criterion may introduce selection bias, potentially excluding relevant studies published in other languages or focusing on other types of cancer	2021
The impact of chemotherapy on cognitive function: a multicentre prospective cohort study in testicular cancer [38]	Multicenter prospective cohort study	*n* = 145	Testicular	No substantive differences in objective or subjective cognitive dysfunction in either group persisted 12–18 months post‐baseline. Patients undergoing chemotherapy for testicular cancer differ from findings in breast cancer populations	Limitations that provide challenges for future studies include the study not being powered to address any difference between the single‐agent chemotherapy	2019
Self‐Reported Cognitive Impairment in Patients With Cancer [39]	Longitudinal Multicenter Study	*n* = 595	Solid tumors	A significant proportion of patients undergoing cancer therapy self‐report problems with memory and concentration. Cognitive problems get worse during treatment and are still in evidence 6 months following the conclusion of treatments	The patients in this study tended to be more educated than the general population and were primarily Caucasian, making this study less generalizable to patients from minority groups or to people with lower socioeconomic status. Also, cancer patients in this study sample were treated as outpatients in community cancer treatment centers and the findings may not be representative of all cancer patients, especially those with more severe or rare cancers that required inpatient hospital care. In addition, we did not have information on whether or not study participants were on antiestrogen or antiandrogen therapy	2007
Chemotherapy‐associated cognitive impairments in Korean cancer patients: Risk factors and functional outcome [40]	Cross‐sectional study	*n* = 163	Breast (27%), Hematologic (33%; leukemia and lymphoma), and Colorectal (18%)	Significant cognitive decline occurred during active chemotherapy; attention to cognitive impairment should be given in the early phase of chemotherapy	Limitations include the heterogeneity of the sample: baseline status (ie, initial treatment and new regimen vs. midcycle), follow‐up time points (i.e., time intervals and the number of treatment exposures between time points), and cancer types	2018
Lower cognitive performance and white matter changes in testicular cancer survivors 10 years after chemothearpy [41]	Cross‐sectional Study	*n* = 51	Testicular	This cross‐sectional study suggests that men receiving CT for TC are at risk for long‐term lower cognitive performance. Although CT affected WM microstructure, this was unrelated to cognitive performance	The sample size is relatively small, which limits the generalizability of the findings. Moreover, there's a potential for selection bias as the consent rate differed between the chemotherapy group (CT) and the surgery‐only group (S‐only). Additionally, the study design is cross‐sectional, which hinders the ability to establish causal relationships between chemotherapy treatment and cognitive impairment. Longitudinal studies would provide more robust evidence regarding the long‐term effects of chemotherapy on cognitive function	2015
Cognitive Impairment in Men with Prostate Cancer Treated with Androgen Deprivation Therapy: A Systematic Review and Meta‐analysis [42]	Systematic Review and Meta‐analysis	32 Articles	Prostate Cancer	Analyses of overall cognitive impairment and use of ADT defined according to ICCTF criteria in a pooled analysis of two prospective cohort studies were inconclusive. The retrospective studies suggested a higher risk, albeit not statistically significant, of overall cognitive impairment after ADT	Better‐designed prospective studies are needed to assess the effect of ADT on cognitive impairment with long‐term follow‐up	2018

The remaining 17 studies specifically examined CRCI in a single cancer type. Five studies focused on colorectal cancer, four on testicular cancer, four on lung cancer, two on prostate cancer, one study on colon cancer, and one study on hematological cancer.

### 3.3 Study Design

The included studies were categorized by design type (see Table 1). The studies utilized the following designs: Longitudinal (*n* = 4), Pilot Study (*n* = 1), Systematic review (*n* = 3), Cross‐Sectional Cohort (*n* = 1), Prospective (*n* = 6), Transition (*n* = 1), Retrospective (*n* = 1), Meta‐Analysis (*n* = 1), Cross‐Sectional (*n* = 7), Case–Control (*n* = 1), Prospective Longitudinal (*n* = 1), Integrative Review (*n* = 1) and Prospective Cohort (*n* = 1).

## 4 Discussion

Cognitive impairment remains a significant concern in cancer treatments, though its severity varies across therapies. While androgen deprivation therapy (ADT) for up to 36 months has not been definitively linked to cognitive decline [39], other treatments, such as chemotherapy and adjuvant FOLFOX4 for colon cancer, have been associated with declines in verbal memory and psychomotor vigilance [14]. A systematic review examining ADT's effects on prostate cancer survivors found no significant risk of overall cognitive impairment in prospective cohort studies. However, cognitive risks differ between treatment types, emphasizing the importance of assessing patients individually.

One of the major concerns is underdiagnosed cognitive impairment, particularly CRCI, which is a significantly reduced quality of life among cancer patients. This issue is particularly pronounced in small cell lung cancer (SCLC) patients, where neuropsychological impairments have been observed both before and after treatment [18]. The cognitive impact of cancer treatments is further influenced by treatment type. Bone marrow transplants and chemotherapy for various cancers, including colorectal and lung cancer, have been linked to cognitive complaints and deficits [22]. However, while some treatments appear to cause only mild and transient cognitive effects, others are associated with more persistent cognitive issues.

Current cognitive assessment tools, such as the Mini‐Mental State Examination (MMSE), lack the sensitivity to detect subtle cognitive changes [43]. This highlights the need for more precise instruments capable of identifying early declines, particularly during the initial phases of chemotherapy. Early intervention strategies may help mitigate cognitive deterioration and improve patient outcomes.

Despite valuable insights into CRCI, several methodological limitations hinder the reliability and applicability of existing research. Many studies suffer from small sample sizes, reducing statistical power and increasing susceptibility to selection and attrition biases. Furthermore, reliance on self‐reported cognitive complaints rather than objective neurocognitive assessments limits the ability to distinguish between subjective experiences and clinically significant impairment. The absence of standardized and validated measures for cognitive function and psychological comorbidities, such as depression and anxiety, introduces recall bias and variability in reported outcomes, complicating cross‐study comparisons.

Study design challenges further constrain the ability to establish definitive conclusions regarding the etiology and trajectory of CRCI. The predominance of cross‐sectional research limits causal inferences between chemotherapy exposure and cognitive decline, highlighting the need for well‐controlled longitudinal studies. Additionally, heterogeneity in patient demographics, cancer types, treatment regimens, and assessment methodologies complicates efforts to generalize findings across oncology populations. These inconsistencies underscore the necessity of harmonized protocols and standardized cognitive assessments to enhance comparability and reproducibility.

Finally, it is important to acknowledge that cognitive impairments observed in cancer survivors may not be solely attributable to chemotherapy exposure. Symptoms such as fatigue, anxiety, depression, and sleep disturbance are highly prevalent among cancer patients and survivors, and these factors can independently contribute to or exacerbate perceived cognitive decline. Several studies have reported significant overlap between these psychosocial and physiological sequelae and CRCI, making it challenging to isolate the direct neurotoxic effects of chemotherapy from the broader cancer experience. Future investigations employing longitudinal designs, pre‐treatment baselines, and biomarkers of neuroinflammation or hormonal dysregulation will be essential to disentangle these complex and interacting pathways.

Addressing these gaps requires a strategic shift toward larger, multicenter longitudinal studies incorporating advanced neuroimaging techniques, objective cognitive testing, and biomarker analyses to elucidate the neurobiological underpinnings of CRCI. Further investigation into the differential effects of specific chemotherapeutic agents, adjunctive treatments, and patient‐specific risk factors will enhance precision medicine approaches to mitigating cognitive decline. Strengthening methodological rigor and integrating multidisciplinary research perspectives will ultimately improve clinical guidelines, optimize survivorship care, and inform the development of targeted interventions aimed at preserving cognitive function in cancer patients.

### 4.1 Recommendations for the Field

Future studies should incorporate more sensitive cognitive assessment tools beyond the MMSE scale to better detect subtle cognitive changes in cancer patients, as traditional methods may lack precision. Since cognitive decline can begin during active chemotherapy, early interventions must be developed and implemented to mitigate its effects, that includes, but is not limited to programs focusing on cognitive rehabilitation, pharmacology, and physical activity. Regular cognitive monitoring, particularly for patients undergoing chemotherapy and bone marrow transplants, is essential. Providing cognitive support and rehabilitation can help manage and alleviate difficulties.

Given the strong association between cognitive dysfunction and depressive symptoms, integrating mental health support into cancer care is crucial. Patients should have access to psychological counseling and interventions to address both depression and cognitive decline. Additionally, improving patient education on the cognitive effects of cancer treatments can help individuals and their families better understand and prepare for potential challenges. Clear and effective communication strategies should be used to inform patients about what to expect and how to manage these changes.

Beyond breast cancer, healthcare practitioners must recognize the broader impact of “chemobrain” on diverse patient populations. Patients should be educated on its risks, ensuring they are well informed and supported throughout their treatment and recovery.

## Author Contributions

**Michael J. Rovito:** conceptualization (lead), formal analysis (lead), methodology (lead), project administration (lead), writing – original draft (lead). **Khushi M. Chauhan:** conceptualization (supporting), methodology (supporting), writing – review and editing (equal). **Humnah Baig:** methodology (supporting), visualization (supporting), writing – review and editing (supporting).

## Conflicts of Interest

The authors declare no conflicts of interest.

## Data Availability

The data that support the findings of this study are available from the corresponding author upon reasonable request.
